# Longitudinal assessment of sNfL and sGFAP in severe NMOSD treated with allogeneic stem cell transplantation

**DOI:** 10.3389/fimmu.2026.1832821

**Published:** 2026-07-01

**Authors:** Barbora Srpova, Eva Krasulova, Libuse Noskova, Michaela Cichrova, Marta Kalousova, Veronika Valkova, Marek Trneny, Vlastimil Kral, Eva Kubala Havrdova, Petra Nytrova

**Affiliations:** 1 Department of Neurology and Centre of Clinical Neuroscience, General University Hospital and First Faculty of Medicine, Charles University, Prague, Czechia; 2 Institute of Medical Biochemistry and Laboratory Diagnostics, General University Hospital and First Faculty of Medicine, Charles University, Prague, Czechia; 3 Institute of Computer Science, Czech Academy of Sciences, Faculty of Mathematics and Physics, Charles University, Prague, Czechia; 4 Institute of Haematology and Blood Transfusion, Prague, Czechia; 5 First Department of Internal Medicine, General University Hospital in Prague and First Faculty of Medicine, Charles University, Prague, Czechia; 6 Health Institute based in Ústí nad Labem, Center for Immunology and Microbiology, Ústí nad Labem, Czechia

**Keywords:** alloSCT, ASCT, NMOSD, sGFAP, sNfL

## Abstract

**Background and objectives:**

Serum neurofilament light chain (sNfL) and serum glial fibrillary acidic protein (sGFAP) in neuromyelitis optica spectrum disorder (NMOSD) have recently emerged as potential biomarkers of disease activity, although their clinical relevance remains uncertain. Our aim was to assess longitudinal sNfL and sGFAP dynamics at the individual level in a patient with a severe course of aquaporin-4-antibody-positive (AQP4-IgG) NMOSD who failed common therapeutic approaches and consequently required autologous (ASCT) and ultimately allogeneic (alloSCT) stem cell transplantation.

**Methods:**

We retrospectively analyzed sNfL and sGFAP levels in 34 longitudinal serum samples collected from a single female patient over an eight-year observation period. Levels of sNfL and sGFAP were measured using the Single Molecule Array (Simoa). Statistical analyses were performed using R software.

**Results:**

The median sNfL was 25.21 pg/mL (IQR 32.74), and the median sGFAP level was 358.03 pg/mL (IQR 1954.06). A positive correlation was observed between sNfL levels and the Expanded Disability Status Scale (EDSS) (r = 0.41, p = 0.0149). Elevated sGFAP levels were associated with clinical relapses.

**Discussion:**

This single-patient study provides unique longitudinal data on sNfL and sGFAP in a highly aggressive course of AQP4-IgG–positive NMOSD requiring allogeneic stem cell transplantation. The findings suggest that sNfL may reflect cumulative disability progression, whereas sGFAP appears to more reliably capture disease activity. These observations should be interpreted cautiously as descriptive and hypothesis-generating only.

## Introduction

Neuromyelitis optica spectrum disorder (NMOSD) is an autoimmune disease of the central nervous system (CNS), primarily considered an astrocytopathy because of its pathognomonic association with antibodies against the water channel aquaporin-4 (AQP4-IgG) expressed predominantly on astrocytes (1). Clinical manifestations typically include relapses of optic neuritis, myelitis, or area postrema syndrome, which often lead to sustained severe disability. Long-term immunosuppressive treatment aimed at preventing relapses is therefore crucial.

We report a longitudinal biomarker dataset comprising repeated measurements of serum neurofilament light chain (sNfL) and glial fibrillary acidic protein (sGFAP) collected over an eight years period in a female patient with treatment-refractory AQP4-IgG-positive NMOSD who underwent high-dose immunoablation followed by autologous (ASCT) and ultimately allogeneic stem cell transplantation (alloSCT).

Several case reports have been published on NMOSD patients treated with ASCT with varying outcomes (2). To the best of our knowledge, only four cases of NMOSD treated with alloSCT have been published so far (3–5).

Given the high risk of severe relapses in patients with NMOSD, reliable biomarkers of disease activity are crucial. Both sNfL and sGFAP are very promising markers in patients with multiple sclerosis (MS) (6). In patients with NMOSD, evidence from clinical trials and small studies have brought important insights, but the applicability of these findings to routine clinical practice is still unclear (7–10).

Therefore, the aim of this study was to descriptively assess longitudinal sNfL and sGFAP dynamics at the individual level in a single patient with a severe course of AQP4-IgG–positive NMOSD who failed standard therapeutic approaches and ultimately required both autologous and allogeneic stem cell transplantation.

## Methods

This was a longitudinal, observational, retrospective study of a single patient, aimed at assessing the clinical course of the disease together with frequent retrospective measurements of sNfL and sGFAP over an eight-year observation period (May 2006, April 2014). Clinical assessments were performed at least every six months or during relapses. Serum samples were collected in accordance with biobanking standards and stored at –80 °C until analysis (11). AQP4-IgG titers were measured during the original clinical follow-up using a commercially available fixed cell-based assay (CBA, Euroimmun, Germany). The levels of sNfL and sGFAP were measured using the Simoa platform (Single molecule array, SR-X, Quanterix, USA, NF-light Advantage kit) retrospectively in 2024 (12). Exploratory statistical analysis was performed using the R software, version 4.5.1. Associations between measurements were assessed using correlation analysis. The Pearson correlation coefficient was used for sNfL-EDSS, whereas the Spearman rank correlation coefficient was used for associations involving sGFAP (sGFAP-sNfL and sGFAP-EDSS) due to the highly right-skewed distribution of sGFAP. A p value <0.05 was considered statistically significant. Because all 34 measurements were obtained from a single patient, the observations are not statistically independent (lag-1 autocorrelations: sNfL 0.59; EDSS 0.36; sGFAP 0.22). Consequently, reported p-values are exploratory only, and 95% confidence intervals for correlations were additionally estimated using a moving-block bootstrap (5,000 resamples; block lengths 3–5) to account for this dependence.

### Clinical context and treatment phases

The analyzed longitudinal biomarker dataset was obtained from a patient with AQP4-IgG positive NMOSD and a highly aggressive disease course. Disease onset occurred in 1998 with optic neuritis. Initial brain MRI was unremarkable and cerebrospinal fluid analysis showed no oligoclonal bands. The diagnosis of NMOSD was confirmed in 2007 using a live cell-based assay for AQP4-IgG, in accordance with the Wingerchuk 2006 diagnostic criteria (13).

Despite escalation through multiple immunosuppressive and immunomodulatory therapies available at that time, including cytostatic agents, anti-CD20 therapy, and a chronic plasma exchange program, recurrent relapses occurred with progressive disability, reaching an Expanded Disability Status Scale (EDSS) score of 6.0.

Given the ongoing disease activity, off-label high-dose immunoablation followed by autologous hematopoietic stem cell transplantation (ASCT) was performed in September 2009. However, clinical disease activity persisted, with recurrent relapses occurring within three months after ASCT. During the subsequent 18 months, nine relapses were documented (annualized relapse rate 6), including a severe relapse associated with incomplete recovery and progression to EDSS 7.0. This period was accompanied by radiological progression on spinal cord and brain MRI, as well as increasing serum AQP4-IgG titers.

Due to continued highly active disease, allogeneic hematopoietic stem cell transplantation (alloSCT) was undertaken as rescue therapy in April 2011 using a nonmyeloablative conditioning regimen. Details of the ASCT and alloSCT protocols are provided below. Following alloSCT, no further clinical relapses were observed. Disability stabilized with partial improvement (EDSS 6.5) and remained unchanged during long-term follow-up. Follow-up MRI demonstrated partial regression and subsequent stabilization of CNS lesions, and AQP4-IgG became undetectable in peripheral blood using a fixed cell-based assay.

**Figure 1:**
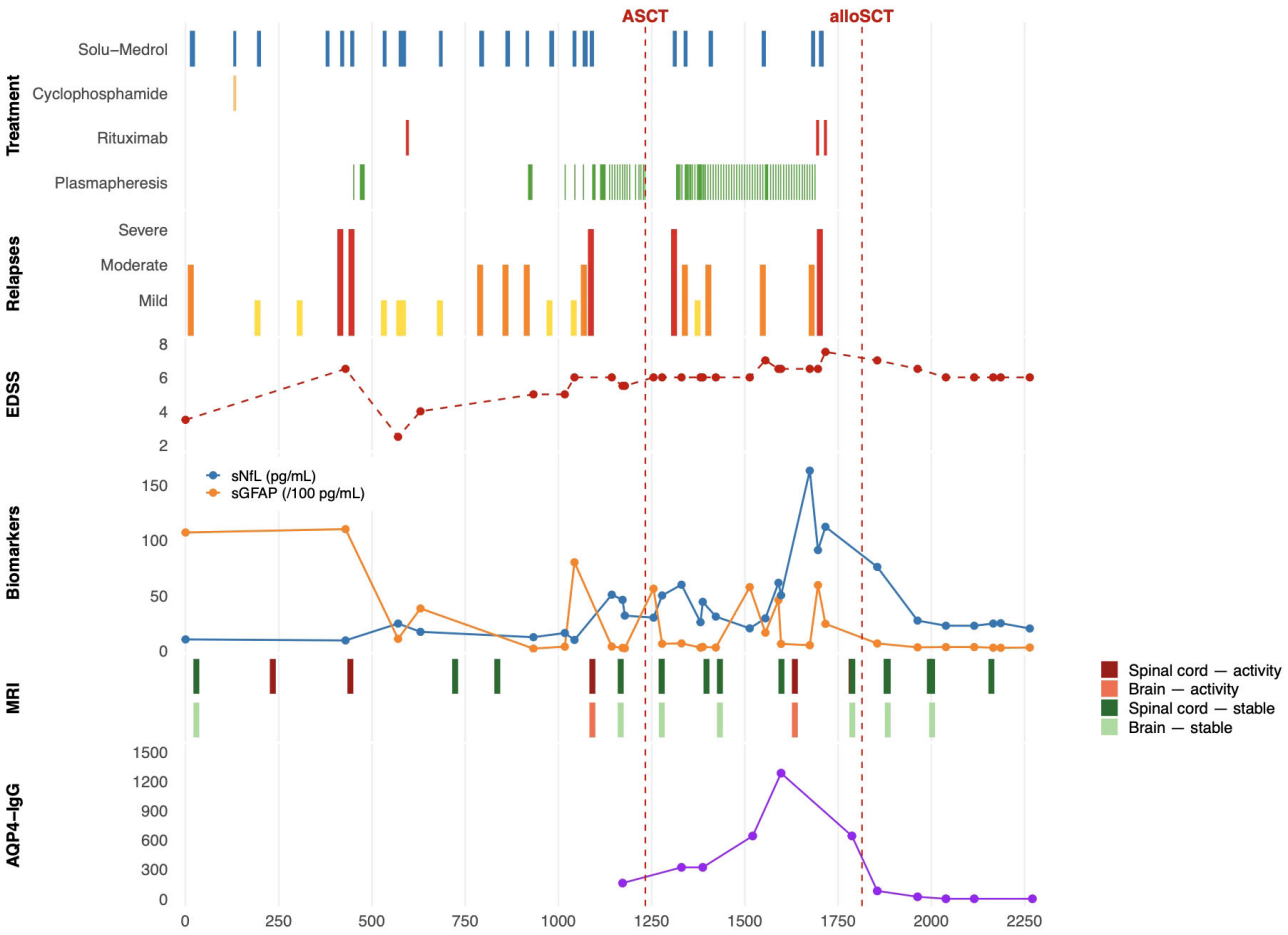
Longitudinal dynamics of sNfL and sGFAP in relation to clinical course, MRI activity and treatment. sNfL, serum neurofilament light chain; sGFAP, serum glial fibrillary acidic protein; EDSS, Expanded Disability Status Scale Score; ASCT, autologous stem cell transplantation; alloSCT, allogeneic stem cell transplantation AQP4-IgG, Aquaporin-4 Immunoglobulin G (titres) MRI stable, no new or enlarging lesions MRI activity, new or enlarging lesions.

Clinical course, treatment modalities, and biomarker measurements are summarized in **Figure 1**.

### Transplantation protocols

#### Autologous stem cell transplantation

Mobilization of CD34+ hematopoietic stem cells was performed using high-dose intravenous cyclophosphamide (total dose 4.5 g), followed by filgrastim (granulocyte colony-stimulating factor, G-CSF; 10 µg/kg/day subcutaneously), initiated on day +2 after cyclophosphamide administration and continued until completion of mobilization. No *in vitro* graft manipulation was performed.

Conditioning was carried out using the BEAM regimen, consisting of carmustine 300 mg/m² on day −6, etoposide 200 mg/m² and cytarabine 200 mg/m² daily from day −5 to day −2, and melphalan 140 mg/m² on day −1. Cryopreserved autologous stem cells were thawed and reinfused on day 0.

Rabbit polyclonal anti-thymocyte globulin (ATG Fresenius) was administered at a dose of 4 mg/kg/day on days +1 and +2 for *in vivo* depletion of autoreactive T lymphocytes. Intravenous immunoglobulins were administered on days +6 and +10 at a dose of 30 g per day.

Early adverse events during ASCT included grade 3 diarrhea and nausea, pancolitis, and sepsis, all of which resolved completely following appropriate antibiotic treatment. No other major adverse events were observed.

#### Allogeneic stem cell transplantation

Allogeneic hematopoietic stem cell transplantation was performed using a non-myeloablative conditioning regimen consisting of fludarabine, cyclophosphamide, and anti-thymocyte globulin (ATG). The bone marrow graft was obtained from an HLA- and ABO-matched unrelated donor.

Graft-versus-host disease (GvHD) prophylaxis consisted of cyclosporine A, mycophenolate mofetil, and ATG as part of the conditioning regimen. In the early post-transplant period, the patient developed grade II acute GvHD involving the upper gastrointestinal tract, skin (affecting up to 50% of the body surface area), and laboratory elevation of liver enzymes. To date, no clinical manifestations of chronic GvHD have been observed.

Stable donor chimerism was achieved.

## Results

The levels of sNfL and sGFAP were analyzed in 34 serum samples. Among these measurements, one was taken during a mild relapse, four were taken within one week before a relapse, and two were taken within one week after a relapse.

**Figure 2:**
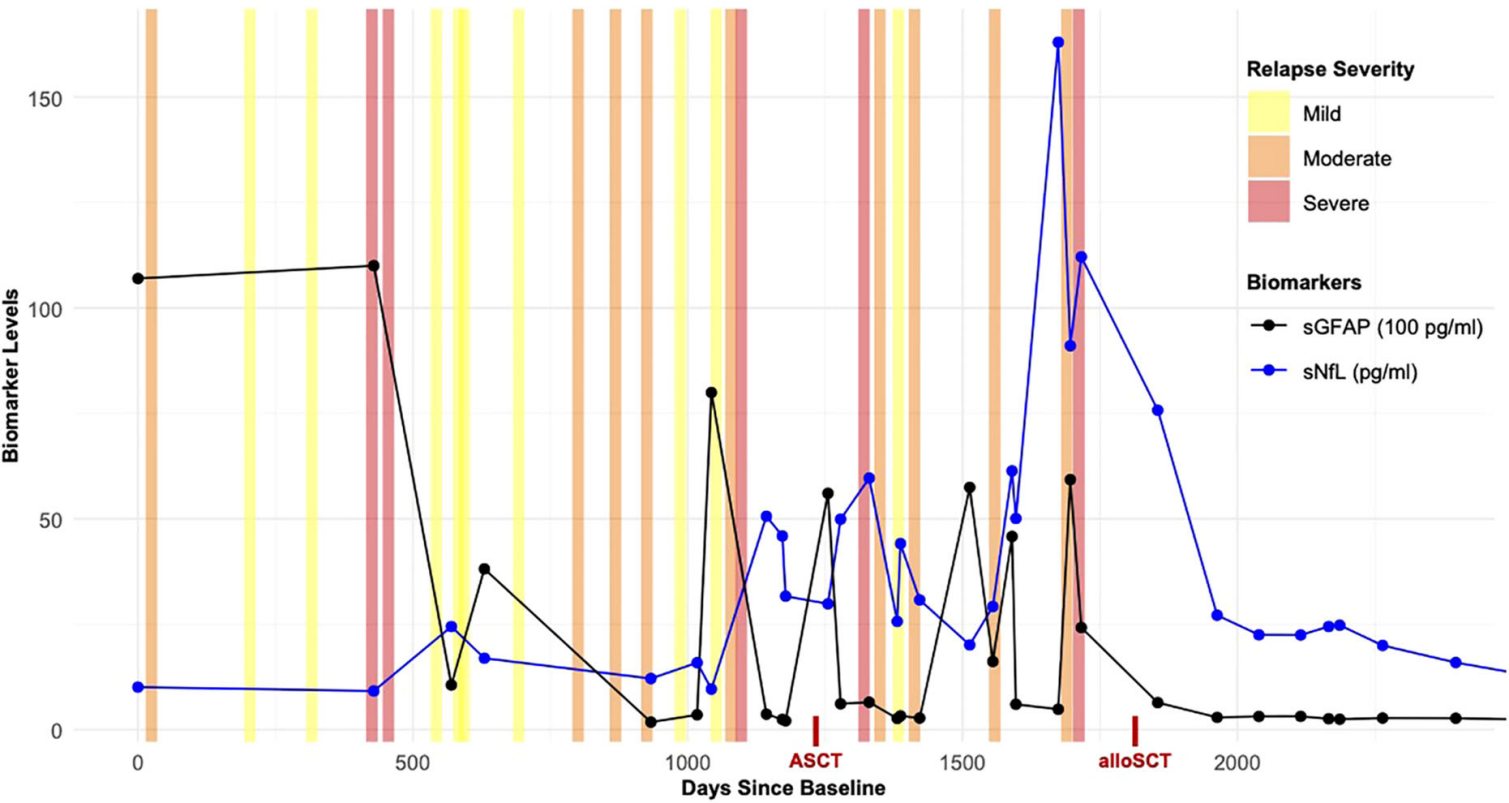
Levels of sNfL and sGFAP in relation to clinical relapses. sNfL, serum neurofilament light chain; sGFAP, serum glial fibrillary acidic protein; ASCT, autologous stem cell transplantation; alloSCT, allogeneic stem cell transplantation.

The median sNfL level was 25.21 pg/mL (IQR 32.74), with a mean of 37.09 pg/mL (SD 32.88; range 9.16-163.02). The median sGFAP level was 358.03 pg/mL (IQR 1954.07), with a mean of 2009.2 pg/mL (SD 3064.87; range 177.73-11002.6). Very high levels of sGFAP, with lower levels of sNfL, were observed at the beginning of the follow-up, while the lowest levels of both sNfL and sGFAP were recorded after alloSCT. A comparison of the development of both markers is shown in **Figures 1** and **2**. No significant correlation was found between the two parameters (ρ = 0.134; p = 0.450; block-bootstrap 95% CI −0.36 to 0.58). The highest recorded sGFAP value occurred during an ongoing relapse. Additionally, increased sGFAP levels were observed near relapses (both before and after), suggesting sensitivity to acute disease activity (**Figure 2**).

**Figure 3:**
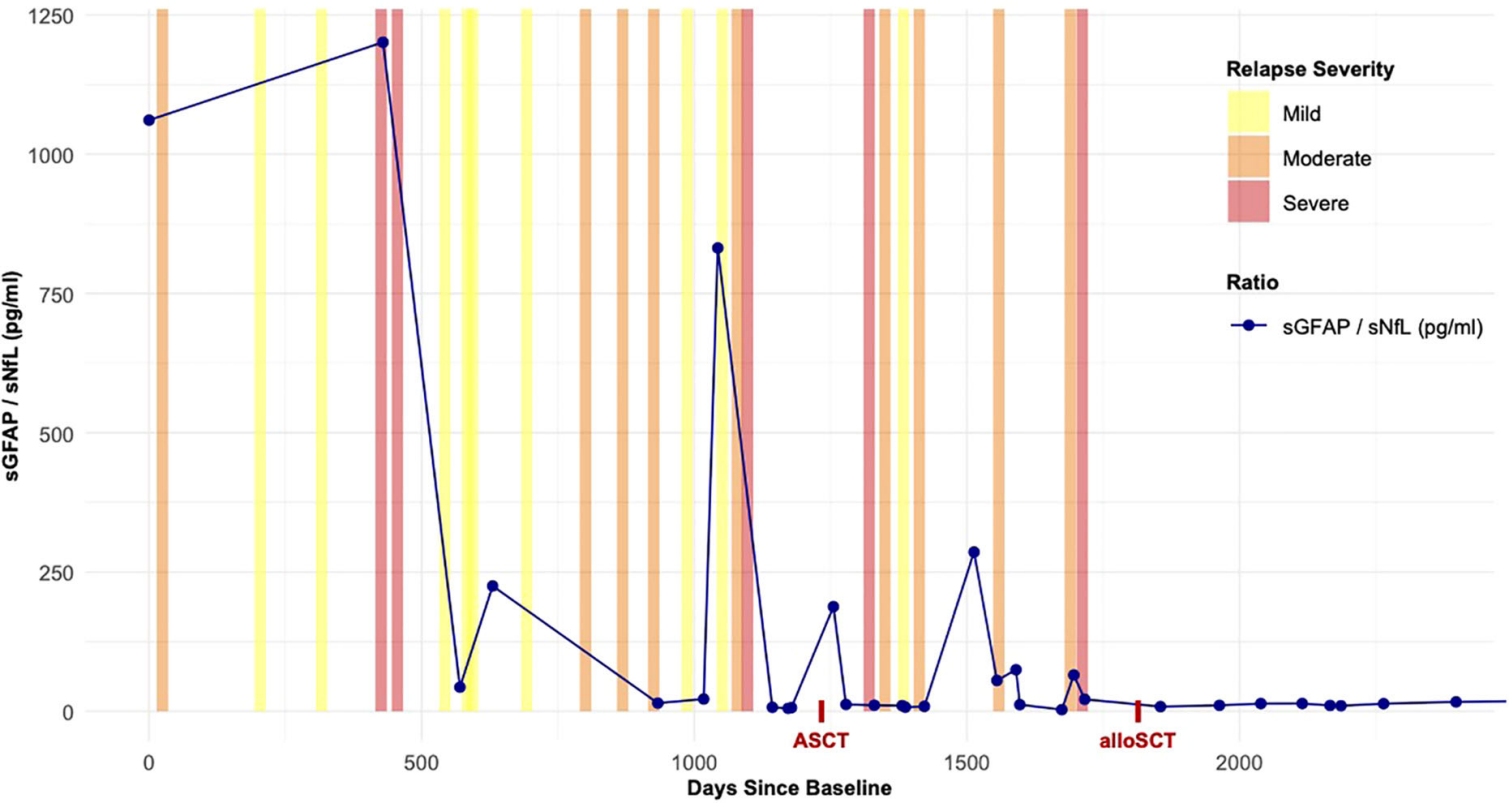
sGFAP/sNfL ratio during relapses and remissions. sNfL, serum neurofilament light chain; sGFAP, serum glial fibrillary acidic protein; ASCT, autologous stem cell transplantation; alloSCT, allogeneic stem cell transplantation.

A positive correlation between sNfL and EDSS was found (r = 0.41, p = 0.0149; block-bootstrap 95% CI 0.12 to 0.66). No correlation was observed between sGFAP and EDSS. The sGFAP/sNfL ratio was highest during the early highly active phase of the disease and decreased over time, with persistently low and stable values observed following alloSCT. Increased ratios were predominantly observed during relapse periods (**Figure 3**).

Additional comparison of longitudinal changes in sNfL and sGFAP with EDSS scores and AQP4-IgG status is provided in **Supplementary eFigure 1**.

## Discussion

This brief report describes a longitudinal, within-subject analysis of sNfL and sGFAP dynamics across different treatment phases in AQP4-IgG NMOSD. Using repeatedly collected biobanked serum samples, we explored temporal relationships between these biomarkers, clinical disability, and disease activity in the context of highly aggressive, treatment refractory disease managed with sequential ASCT and alloSCT (14). In this setting, alloSCT was undertaken following insufficient disease control after ASCT and was associated with sustained clinical and radiological stability.

The effect of alloSCT is consistent with the proposed concept that this procedure replaces the autoreactive host immune system with a new, presumably tolerant, donor-derived immune system. In contrast to ASCT, the conditioning regimen in alloSCT serves not only to eradicate autoreactive effector cells but also to enable durable engraftment of donor hematopoiesis (15).

Given the very severe disease course of NMOSD, the rare treatment approach, and the frequent biobanking of serum samples, we performed a retrospective assessment of sNfL and sGFAP levels. The observed sGFAP concentrations in our patient were within the range reported in previous NMOSD biomarker studies, including highly active disease phases described in the N-MOmentum study. In the same study, an sNfL cut-off of 32 pg/mL measured during attacks was reported to distinguish patients with subsequent EDSS worsening from those without disability worsening at follow-up. In our patient, several sNfL values exceeded this threshold during the highly active disease phase, which may be consistent with cumulative neuroaxonal injury and disability progression. However, given the single-patient design and the descriptive nature of our analysis, this threshold cannot be validated in our dataset and should therefore be interpreted only as a contextual reference (16). A correlation between both biomarkers was not found. The sGFAP levels were more fluctuating throughout the follow-up and increased during relapses. On the other hand, sNfL showed a correlation with EDSS score and overall disability, but the association with relapses appeared less pronounced than for sGFAP. These findings are consistent with previous studies suggesting a potential role of sGFAP as a biomarker of disease activity, but they partially contrast with the work of Watanabe et al., as we did not observe a correlation between sGFAP and EDSS (7, 10, 17). On the other hand, the longitudinal dynamics of the sGFAP/sNfL ratio may support previous observations by Watanabe et al., suggesting that relatively higher sGFAP/sNfL ratios reflect predominant astrocytic injury in NMOSD, particularly during active disease phases. In our patient, the highest sGFAP/sNfL ratios were typically observed before severe relapses during the early highly active phase of the disease, whereas persistently low and stable values were observed following alloSCT (10). Furthermore, sNfL trajectories appeared to mirror AQP4-IgG titers across the available timepoints (**Supplementary eFigure 1C**), with both rising during the period of progressive disease activity and declining to low or undetectable values after alloSCT. This pattern would be consistent with sNfL reflecting downstream neuroaxonal injury secondary to AQP4-IgG–mediated astrocytopathy, although the limited number of AQP4-IgG measurements precludes a formal quantitative comparison.

Our study is limited by its retrospective single-patient design. An additional important limitation is the highly complex treatment course, which may have influenced the observed biomarker dynamics. Repeated plasma exchange procedures may have influenced circulating sNfL and sGFAP concentrations, as previous studies suggested that apheresis itself can alter serum biomarker levels independently of disease activity. Both sNfL (~68 kDa) and sGFAP (~50 kDa) are proteins of sufficient molecular size to be directly removed from the intravascular compartment during plasma exchange procedures, and albumin replacement may further contribute to transient lowering of measured serum concentrations through dilutional effects. In addition, redistribution between intravascular and extravascular/CSF compartments may further influence post-apheresis biomarker kinetics (18, 19). In our patient, blood samples for biobanking were consistently obtained prior to plasma exchange procedures. However, during periods of very frequent plasma exchange (approximately once weekly), a potential influence of apheresis on circulating biomarker levels cannot be fully excluded. To address the non-independence of repeated measurements within a single patient, a moving-block bootstrap sensitivity analysis was performed. The resulting confidence interval was consistent with the parametric estimate.

Taken together, these findings raise the possibility that sGFAP may be a more sensitive marker of disease activity than sNfL in NMOSD. Beyond demonstrating sustained disease stability following alloSCT in a highly aggressive NMOSD, this report highlights the added value of individualized longitudinal assessment of serum biomarkers, particularly sGFAP.

## Data Availability

Anonymized data not published within this article will be made available by request from any qualified investigator. Requests to access these datasets should be directed to barbora.srpova@vfn.cz.

## Supplementary material

Supplementary Figure 1 Longitudinal relationships between sNfL, sGFAP, EDSS, and AQP4-IgG titres. sNfL, serum neurofilament light chain; sGFAP, serum glial fibrillary acidic protein; EDSS, Expanded Disability Status Scale Score; ASCT, autologous stem cell transplantation; alloSCT, allogeneic stem cell transplantation; AQP4-IgG, Aquaporin-4 Immunoglobulin G
