# Supplementary figures and images for: Longitudinal assessment of sNfL and sGFAP in severe NMOSD treated with allogeneic stem cell transplantation
Source: Front Immunol. 2026 Jul 1;17:1832821. doi: 10.3389/fimmu.2026.1832821 (PMC13368567; doi:10.3389/fimmu.2026.1832821)

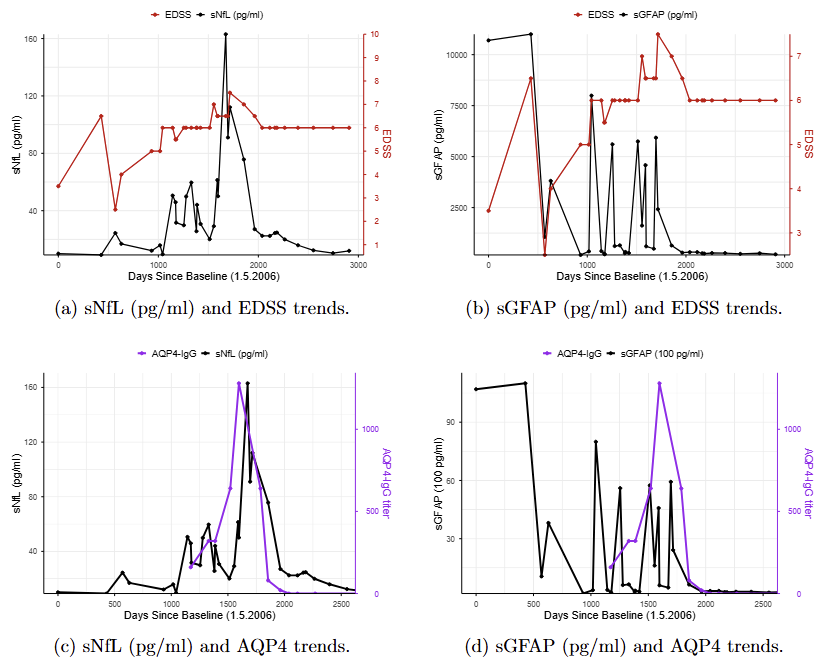

Supplement: Supplementary Figure 1 — Longitudinal relationships between sNfL, sGFAP, EDSS, and AQP4-IgG titres. sNfL, serum neurofilament light chain; sGFAP, serum glial fibrillary acidic protein; EDSS, Expanded Disability Status Scale Score; ASCT, autologous stem cell transplantation; alloSCT, allogeneic stem cell transplantation; AQP4-IgG, Aquaporin-4 Immunoglobulin G [file Image1.png]
